# Socio-economic pandemic modelling: case of Spain

**DOI:** 10.1038/s41598-023-44637-y

**Published:** 2024-01-08

**Authors:** Jan E. Snellman, Nadia L. Barreiro, Rafael A. Barrio, Cecilia I. Ventura, Tzipe Govezensky, Kimmo K. Kaski, Maarit J. Korpi-Lagg

**Affiliations:** 1 Department of Computer Science, Aalto University School of Science, 00076 Aalto, Finland; 2 Instituto de Investigaciones Científicas y Técnicas para la Defensa (CITEDEF), 1603 Buenos Aires, Argentina; 3 Instituto de Física, Universidad Nacional Autónoma de México, 04510 CDMX, Mexico; 4 (CONICET) Centro Atómico Bariloche-CNEA, 8400 Bariloche, Argentina; 5 Universidad Nacional de Río Negro, 8400 Bariloche, Argentina; 6 Instituto de Investigaciones Biomédicas, Universidad Nacional Autónoma de México, 04510 CDMX, Mexico; 7 The Alan Turing Institute, 96 Euston Rd, Kings Cross, London, NW1 2DB UK; 8 Max-Planck-Institut für Sonnensystemforschung, Justus-von-Liebig-Weg 3, 37077 Göttingen, Germany; 9 Nordita, KTH Royal Institute of Technology, Stockholm University, Hannes Alfvéns väg 12, 11419 Stockholm, Sweden

**Keywords:** Complexity, Computer modelling, Dynamical systems, Nonlinear dynamics, Numerical simulations, Computational models, Socioeconomic scenarios

## Abstract

A global disaster, such as the recent Covid-19 pandemic, affects every aspect of our lives and there is a need to investigate these highly complex phenomena if one aims to diminish their impact in the health of the population, as well as their socio-economic stability. In this paper we present an attempt to understand the role of the governmental authorities and the response of the rest of the population facing such emergencies. We present a mathematical model that takes into account the epidemiological features of the pandemic and also the actions of people responding to it, focusing only on three aspects of the system, namely, the fear of catching this serious disease, the impact on the economic activities and the compliance of the people to the mitigating measures adopted by the authorities. We apply the model to the specific case of Spain, since there are accurate data available about these three features. We focused on tourism as an example of the economic activity, since this sector of economy is one of the most likely to be affected by the restrictions imposed by the authorities, and because it represents an important part of Spanish economy. The results of numerical calculations agree with the empirical data in such a way that we can acquire a better insight of the different processes at play in such a complex situation, and also in other different circumstances.

## Introduction

The Covid-19 pandemic has turned out to be one of the major global hazards of the 21st century, causing almost 7 billion infections and nearly 8 million deaths around the world^1^. This unpredictable phenomenon has resulted in substantial changes in our lives, such as the way we act, react, and work, not to mention its impact on our mental health, personal fears and the way we relate with each other. Over the past 3 years, we have experienced dramatic social changes, in particular the way we respond to the restrictions imposed by the governmental and local authorities to maintain the society functioning as normally as possible. However, people tend to act primarily according to their own immediate worries, well being and health. But their reactions may change rapidly due to getting real time information of the status of the pandemic, globally and locally. Moreover, people’s reactions are guided by their personal attitude, the local environment and cultural habits. By now it is clear that we are facing a very complex societal system in which the interaction of many variables can change its dynamics in unexpected ways. Therefore, to model such a system, for getting insight into the processes in play, there is need to simplify it by retaining only those variables that we consider relevant together with introducing stochastic mechanisms.

Here we are interested to model what impact the pandemic could have on the economic performance of a country. There are a number of sources of economic data in the literature (Eurostat, OECD, Our World in data^2^, etc.), upon which we could do data-driven modeling, as there is trustworthy quantification of the number of contagions and deaths caused by Covid-19. In the context of the COVID-19 pandemic, the economic effects of the governmental mitigation measures and relief efforts have been the focus in multiple studies, see for example^3–5^. There are various models that have recently been developed to deal with the dynamical spread of the disease over a geographical region, (see for instance^6–11^), and also studies about the behaviour of several economic indexes during hazardous times^12^. However, only a few studies take into account both, the economic performance and the spread of the pandemic simultaneously, which is the problem we focus on here.

In 2020, Inoue and Todo^13^ applied an agent based model to predict the negative economic effect of a major city (Tokyo) lockdown on preventing the Covid-19 spread, by quantifying the total country’s production losses due to the propagation of economic impacts of lockdown through the supply chains. Inspired by the previous work on the economic response to natural disasters, Pichler et al.^14,15^ proposed an out-of-equilibrium data-driven dynamic input-output macroeconomic model, to address the suddenness and severity of the first wave of the pandemic effects on the demand and supply chains of different sectors of the UK economy. They also consider demand shocks to consumption due to “fear of infection” and “fear of unemployment”. This agent-based model for UK was developed during March–April 2020, and used to forecast the economic consequences of lockdown relaxation by July 2020. Recently we have published a model that combines the geographical spread of the disease and the social changes triggered by it^16,17^, but we have not tested it against a real situation.

We have chosen Spain as the country of interest, since there are accurate monthly data available of the progress of the pandemic during the last 3 years and the data also contain several economic indices (Eurostat, 2023). We have considered that the main non-pharmaceutical measures taken by the government have to do with the restrictions that affect the mobility of people, either by confinement at home, closing schools and shops, or limited traveling. Therefore, we have chosen to follow the behaviour of a particular aspect of the economy that has to do directly with people’s mobility and travelling, which is the number of rooms occupied in the tourist industry. As it can be seen in the Eurostat data, most of the other important aspects of the economy were not greatly affected by the pandemic, and others even increased during these times (such as the pharmaceutical industry, with the massive production of vaccines).

Our next simplification is that we model the geographical spread of the disease by considering stochastic processes that depend on mobility parameters for people’s terrestrial or aerial moves. Social reaction to pandemics determines the mobility values of people. A further simplification is that we take into account only three main concerns of the authorities and the populations, namely, their worries about the economy, the health and the constraints in their activities.

In the next section, we explain the model leaving the details to the Supplementary Material, followed by the analysis of the data used for the pandemic and for the economic tourism index. Then, in “Data” and “Model fitting”, we explain the application of the model to the specific case of Spain and the way the parameters of the system are adjusted by taking into account the restrictions of mobility and vaccination methods implemented. In “Comparison of numerical simulations with real data”, we present the results of the model calculations and discuss the relevant information obtained by them and compare them with other interesting recent results^18^. Finally, we present our conclusions.

## Hybrid epidemic and socio-economy model

**Figure 1:**
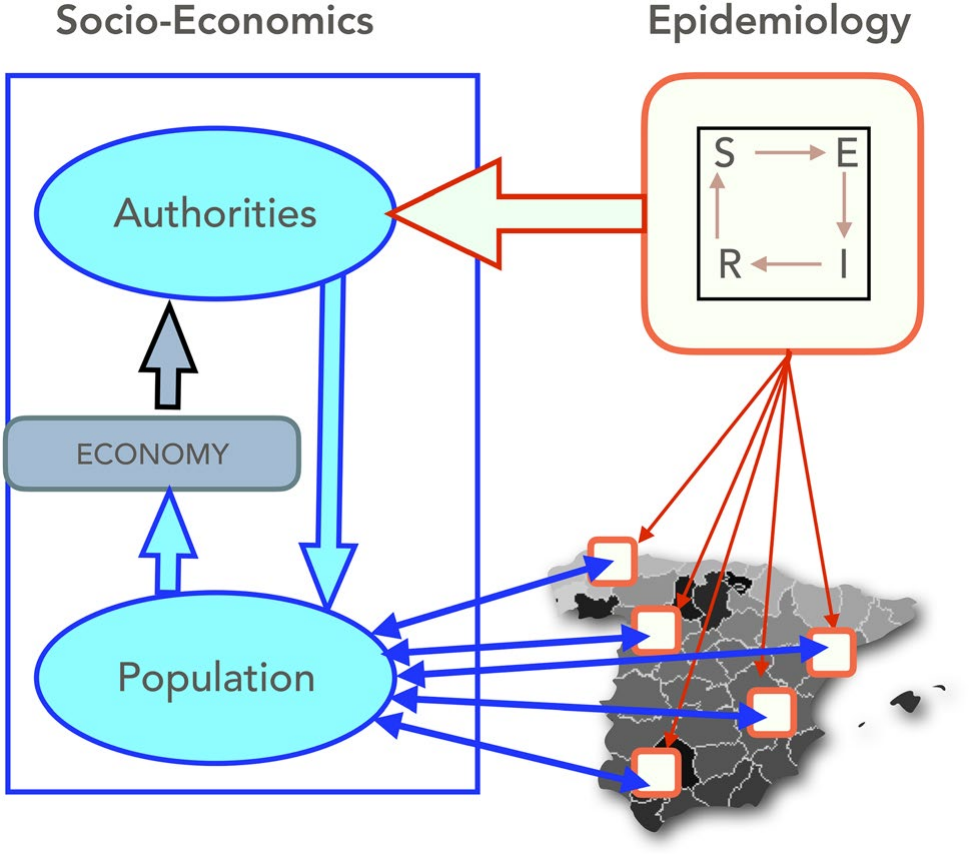
Description of the model. One epidemiological BTH-SEIRS model is defined in each cell of a geographic area (thin red arrows) and each provides information about the proportion of infected people used as input in the socio-economic model (yellow arrow). Authorities and population agents interact taking into account infection level, economic situation and compliance to authorities, as a consequence, agents modify their mobility, which is used to determine the geographical spread in the epidemiology model (thin blue arrows).

In this study, we aim to model the COVID-19 pandemic and its socio-economic effects in Spain using the hybrid BTH-SEIRS model presented in^16^. This is the first time that the model is applied to a realistic situation, and as such we have made some necessary modifications as well as some additions suggested by our other recent works^9,11^, such as implementations of vaccinations and virus strains. The structure of the model is illustrated in Fig. 1, where the different aspects of the model are shown: the authorities of the Spanish provinces and local populations are modeled by the agent-based BTH method^19–21^, while the epidemiology of the COVID-19 pandemic is modeled by a compartmental SEIRS model in which the epidemic spreads from one location to another according to the mobility of the modeled populations. These populations react to mitigate the spreading epidemic by reducing their economic activity, which also reduces their mobility. The growing epidemic also affects the modeled authorities, which can respond by putting up restrictions to the populations in order to reduce their mobility, which in turn affects the propagation of the epidemic. In addition to the severity of the epidemic, the authorities also take into consideration the effect that the actions of the populations have on the total economic activity in their areas, and modify their restrictions according to their preferences. In the next two subsections we describe the model on a general level, and give its exact details in the Supplementary Material.

### Agent-based socio-economic features

The BTH-SEIRS model used in this study is a hybrid model incorporating agent-based, compartmental, and geographical features. In the latter sense, the model concerns a region (in this case the country of Spain), which is subdivided to smaller districts (provinces of Spain). The districts themselves are subdivided to much smaller cells, which form the basic geographic unit observed in the model. The agent-based features of the model center around the populations of the geographic cells and the authorities of the larger districts that are modeled as different types of agents with different roles, named population and authority agents, respectively. The population agents can lower their economic activity in an effort to mitigate the epidemic, while the authority agents can make recommendations to the population agents in their districts on how much the economic activity levels should be lowered at minimum for the same goal.

The idea behind the BTH method is that the main motivation of people is to attain superior social position in comparison to others. We have chosen this approach because it allows for modelling the values of the agents: in order to establish relative social positions between a group of agents, there must naturally exist one or more measures on which the agents can be compared against each other. According to the BTH principle the agents will value the measures on which they rank highly, and likewise devalue the measures on which their ranking is low. Thus in the BTH method the values of agents emerge as weights they assign to measures that define their social position. In the context of the COVID-19 pandemic this allows us to model the social reactions to the deteriorating health situation and to the governmental restrictions, along with attitudes to social responsibility. All of these concerns have been featured extensively in the public conversations about the pandemic^22–25^, and thus they are of prime interest in the social side of the model.

The behaviour of the population agents is defined in our model by how much they value their own economic contribution to mitigating the pandemic, keeping the infection rates low and their own compliance to the restrictions that the authority agents have put in place. Similarly, the authority agents value their own contribution to the pandemic mitigation in the form of introducing restrictions, attempting to keep the rate of infections low while trying to preserve economic activity in their districts. Considering the social interpretations of these values, the desire to keep infection rates low is self-explanatory in the context of a pandemic, while the desire to contribute to the pandemic mitigation in one way or the other can be attributed to social responsibility. The value that the population agents put in compliance can be understood either as the strength or weakness of cultural norms (see, e.g.^26^) or how much respect the populations have for the authority in general, or a combination of these two factors. The authority agents’ valuing of economic activity can be viewed as resulting from the fact that even in so-called normal times the economic situations of countries and their sub-regions are often used to judge their performance and rank them relative to others. Hence it makes sense that these concerns would play a role in the challenging economic circumstances of the pandemic. It should be noted that the population agents have knowledge of their own situation concerning the local infection rate, local mitigation efforts, and compliance as well as the situation of their immediate neighbours. On the other hand all the government agents have the corresponding knowledge of their own situation concerning the regional infection rate, restrictions, and economic activity reduction as well as that of all the other government agent. In this way, they can react to the pandemic in a limited manner even when it has not yet spread to their respective territories. Empirically, such a self awareness by real human populations has been observed in^27^.

In order to simplify the decision making algorithm of the agents in our model, we have assumed that the agents only reluctantly attempt to mitigate the epidemic while recognising that the mitigation is necessary to avoid even worse outcomes. This allows the agent action to vary more dynamically according to the simulated epidemic, but rules out certain approaches that some governments took during the COVID-19 pandemic. Most notably the herd immunity strategy, in which the disease is allowed to spread in an uninhibited manner so that the population acquires the immunity to it naturally, is inconsistent with this premise. The herd immunity approach, however, was not favoured by most countries of the world, with Sweden possibly being the most significant exception (see^28^). Most countries instead tried to balance the mitigation measures and the severity of their socio-economic effects with the health effects of the pandemic, which is consistent with our assumptions^29,30^.

In order to keep our model as simple as possible within the BTH framework, we have sought to include only the relevant social factors. Much empirical research has been done to study the social aspects of the COVID-19 pandemic from various perspectives, see a summary^31^. In recent studies such as^32,33^ the authors have explored the relation between the public attitudes and knowledge and the adoption of various non-pharmaceutical interventions (NPIs), along with things like the effects of risk perception. In another study^34^ the authors take a more general look into the socio-economic determinants of mobility during the pandemic. Social factors such as these could be implemented in the BTH-SEIRS model at the cost of hugely complicating the model with opinion formation dynamics, or allowing the agents consider multiple future possibilities and the associated risks, but we relegate these extension possibilities to future work.

### Epidemiological features

The spreading of the epidemic in the model is governed by SEIRS mechanics first introduced in^35^: the simulated epidemic starts in a single geographical cell and spreads to other neighbouring or distant cells at random which in the BTH-SEIRS model has been modified to being dependent on agent action. For each basic geographical unit, a compartmental SEIR system which includes vaccines and simplified variants is solved. Additionally, different propagation mechanisms between cells that depend on mobility $\nu$ are implemented (see the Supplementary Material). As a rule, the more the population agents lower their economic activity, the lower the chances of the epidemic spreading between the cells. Strains have been implemented by letting the SEIRS transmission rate, i.e. the rate with which the number of infected grows in each cell varies according to different variants.

## Data

**Figure 2:**
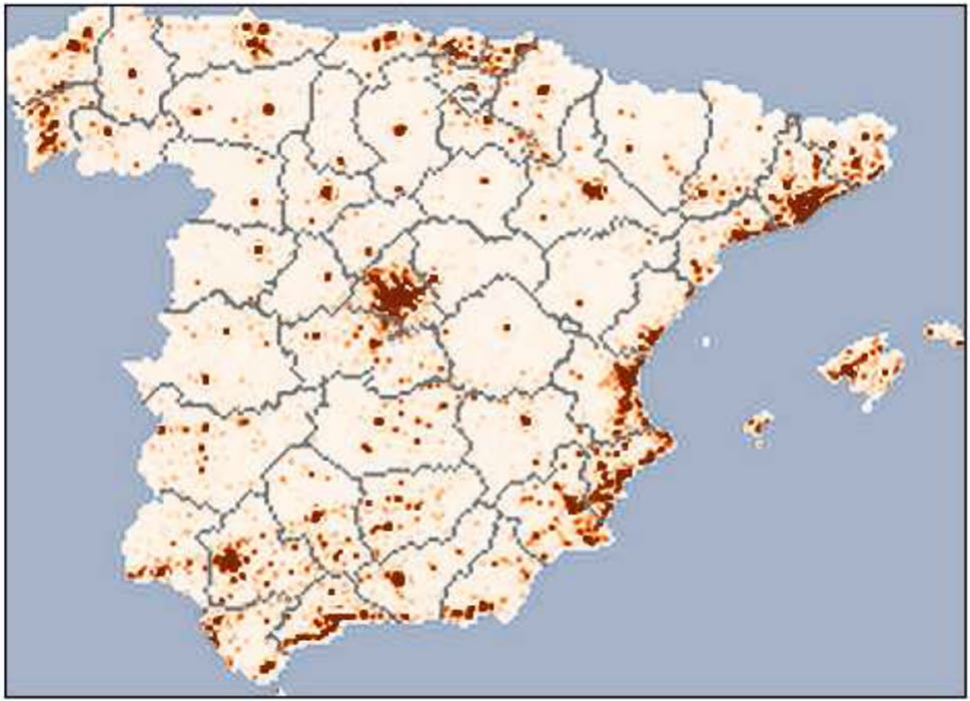
The map of Spain used in the simulations. The different shades of red show the normalized population density distribution. Provinces are separated by grey lines. Information obtained from worldpop.org^36^.

In this study, we compare our modeled infection rates to hose reported in Our World in Data^2^. We also use stringency index data from^30^ to form a general picture of the social response to COVID-19 for the different periods of the pandemic in Spain, and adjust the model accordingly. These adjustments are discussed in detail in “Model fitting”. As for the geographical data, the model uses the population density data from worldpop.org^36^, which is illustrated in Fig. 2. As a comparison to the modeled economic activity we use the Eurostat data on the nights spent at tourist accommodation establishments per each month. Why we use this particular type of economic data deserved a detailed explanation, which we provide next.

The levels of economic activity that the BTH-SEIRS model gives rise to, are not directly related to any existing economic data type that is widely collected. Therefore, we need to use reasonable proxy data in order to facilitate comparisons with the real COVID-19 pandemic in Spain. Ideally, the modelled economic activity should correspond to the time people use for activities involving close social interactions between people, such as shopping, travelling, or attending sport or musical events. These, in turn, can increase disease transmission rates. While this type of data has been gathered for the COVID-19 pandemic in the context of time-use research, the temporal resolution of these studies is not dense enough for our purposes. For example, the UK study^37^ involved the results of only five surveys during the pandemic times, which is very low number considering that we would like to have quarterly or even monthly data for our purposes.

The usual economic indicators that are recorded at monthly or quarterly intervals, most prominent of which is the gross domestic product (GDP), are problematic in other ways for our purposes. Firstly, many of them, especially the GDP, are monetary measures that do not directly tell us anything concerning the behaviour of people. Secondly, they even tended to grow during the pandemic, which is in direct contradiction of the design philosophy of BTH-SEIRS model. As mentioned above, the economic activity level in the model represents time that people use for economic activities, which bring them to close contact with others. While economic growth as a concept explains the ever rising GDP, it is not applicable to the time that people have at their disposal. Otherwise, people would eventually be using more than 24 h a day for economic activity. It is known that GDP is also affected by the economic relief offered by the governments^4^, which we have not taken into account in our model at all. This is a further reason why the economic activity levels obtained from our model cannot be expected to correlate with GDP.

In the end, we found that the most convenient source of data for our purposes is the data concerning the tourist visits in Spain. This sort of data is available from the Eurostat, and we specifically use the “Monthly nights spent at tourist accommodation establishments” statistic as a proxy for the economic activity levels in the BTH-SEIRS model, as it is directly related both to the spreading of the pandemic and economic activity. Another reason to choose this kind of data is that it provides a good measure to compare the socio-economic and epidemiological parts of the model, connected through the mobility, which strongly affects tourism.

**Figure 3:**
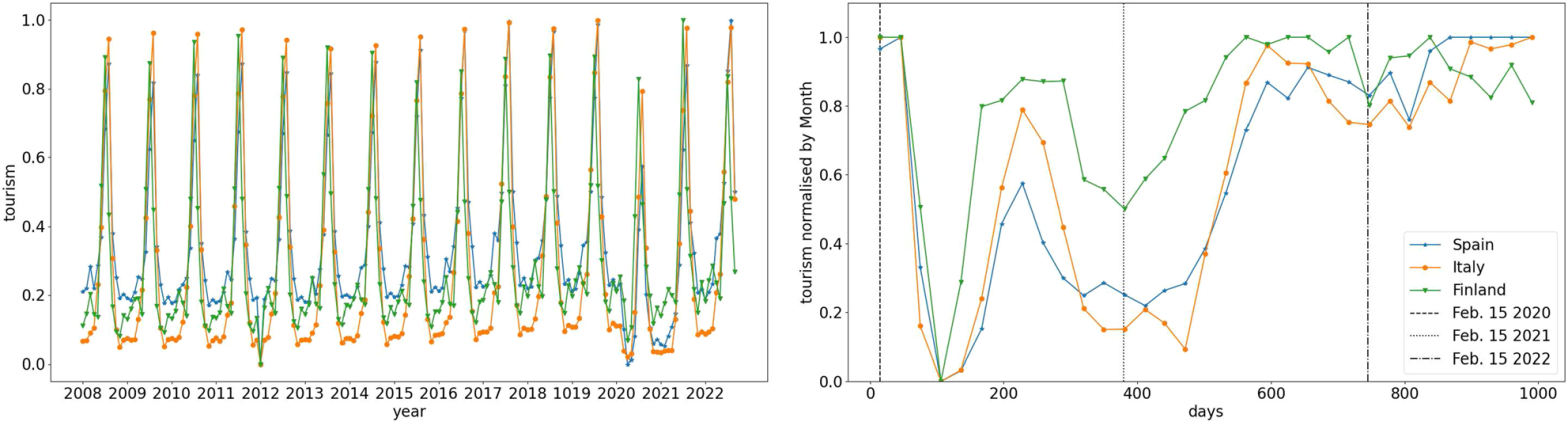
Monthly nights spent at tourist accommodation establishments in Spain, Italy and Finland (left panel) and the economic activity proxy based on this data (right panel). Source: Eurostat.

The data are shown in Fig. 3: the left hand panel shows the raw tourist visit data normalised by the maximum value, and the right hand panel shows the same data for the pandemic times adjusted to the range of [0,1] by removing the global minimum and normalising the result with monthly maximums in order to account for the seasonal nature of tourism visible on the left hand panel. For the sake of comparison, we show the data handled the same way from Finland and Italy along with the data from Spain.

The left hand panel of Fig. 3 starts from the year 2008, which is notable considering that it contains data from the times of the 2007–2008 financial crisis. As can be seen, the financial crisis had little, if any, effect on tourism when compared with the effects of the pandemic. This confirms the obvious fact that the tourism sector is very vulnerable to shocks that affect people’s mobility, especially pandemics. In the end of the timeline we find that the 2022 invasion of Ukraine by Russia similarly does not seem yet to have caused a significant disruption to tourism.

To further validate the meaningfulness of using the tourism as a proxy of economic activity, we have used the same method for Finland and Italy, and show the results in the right panel of Fig. 3. This shows that the proxy has very similar trends in all of the countries, while there are obviously some quantitative differences, especially when Finland is concerned. As Spain and Italy are both Mediterranean countries with similarly profiled tourist sectors, similar trends can be expected for them. The common trends experienced by the countries can be listed as follows:

1. A very steep decline at the beginning of the pandemic ending around May and June of 2020.
2. A period of rapid recovery right after the crash until about September or November of 2020.
3. After November 2020 the economic activity proxy falls again, but not as sharply as in the initial phase of the pandemic, and does not sink quite as low.
4. Around May 2021 the economic activity proxy starts climbing again, gradually reaching the levels prior to the pandemic.

In the last phase, there is some variance between countries in the timing of the normalization, and for a time both Italy and Spain are trending just below normal levels, but by May 2022 at latest they are all behaving as one would expect considering the prepandemic data. In any case, the conclusion that can be drawn from Fig. 3 is that the effects of the COVID-19 pandemic are easily observed from the tourism data, and therefore it makes sense for us to make a proxy for economic activity based on tourism data.

Many of the other Eurostat data sets that show a great disruption due to COVID-19 pandemic are also tourism related, such as the number of arrivals at tourist accommodation establishments and air passengers. While these could be used to create a composite proxy with the data we are using, we choose to use the data on nights spent at tourist accommodation establishments for the reason stated above: because it is much better tied to the time people spend on an economic activity related to their mobility. Of the economic measures that are not directly tied to tourism but are significantly affected by the pandemic, Eurostat data on monthly volume of retail trade is the most relevant for our purposes. This is because retail trade may involve people coming into close contact with each other in shopping centres. However, retail trade may also be affected by factors that are pandemic related but not in any way taken into account in our model, such as inflation. Eurostat data shows that inflation has grown in Spain and EU in general significantly in the latter half of the acute pandemic period even before the Russian invasion of Ukraine in 2022, which may have discouraged people from spending money in retail trade. In the case of Spain, for example, we see that while the pandemic caused a shock decline in the retail trade, the levels of the trade never fully recovered from the hit, staying stubbornly at a stable but lower level than before the pandemic. As this might be an effect caused by inflation, we have chosen not to consider retail trade in our economic proxy.

## Results

In this section, the simulated infection rates and the economic activities obtained from the BTH-SEIRS model are compared with the real world data from Spain, hit by the COVID-19 pandemic. More specifically, we aim to reproduce the tourism based proxy constructed from the Eurostat data as detailed in the previous section, while only drawing some comparisons with the infection rate data from our world in Data^2^. The epidemiological parameters of the model are set according to our earlier work, while the socio-economic parameters are treated as free. Our task is then to find a set of socio-economic parameters such that the economic activity proxy is replicated as well as possible. This section contains two subsections, first of which details the process of finding the suitable parameter values, and the second which concerns the actual comparison of the simulated and real data.

### Model fitting

The BTH-SEIRS model has both epidemiological and social parameters. The former parameters were set according to our former publication^11^, taking into account the virulence of different virus variants and the rate of population immunization. This work seeks to understand the social and economic dynamics of the population in response to the pandemic, therefore we do not attempt to replicate the curves of the number of cases. Furthermore, it should be considered that such daily case curves provide a guide to establish the approximate number of cases but can be incomplete or inaccurate, given that not all people who contracted the disease were tested, or if they did, they generally did not report it on the same day they got sick. Keeping this in mind, we use the daily cases obtained from the SEIRS model only as a source of information for the decision-making mechanisms. The BTH model has to reflect the economic proxy data from Spain. In order to do so, we took the following considerations into account.

**Figure 4:**
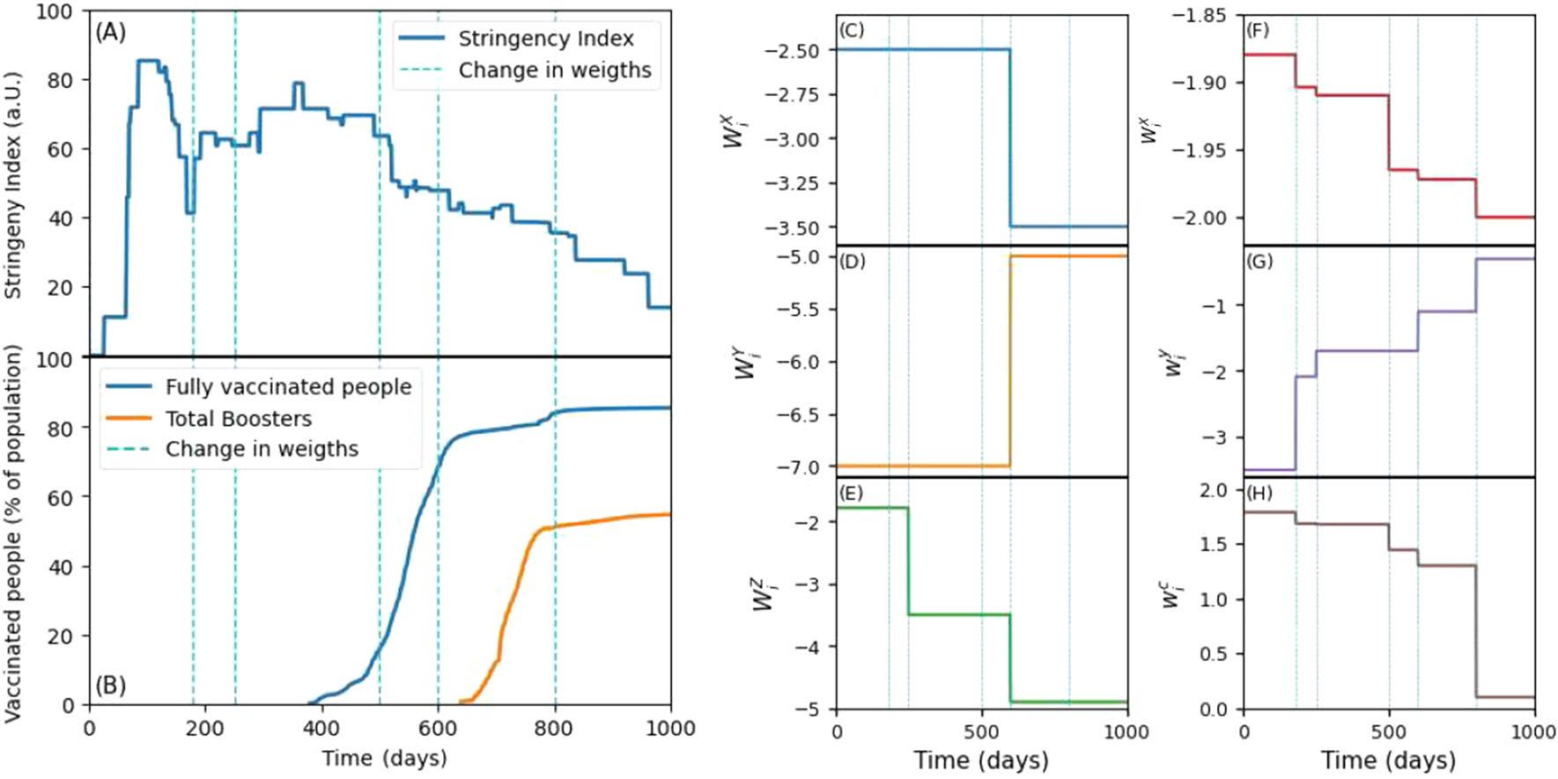
Information used to help choosing dates to change weights and fitted values. (**A**) We show the stringency index^30^ as a measure of how strict were the measures taken by the government. (**B**) Vaccination percentage is shown. The blue line represents total vaccination. Orange line accounts for vaccine boosters^2^. In both figures vertical dashed lines represent the moments we chose to change the weights in the model. (**C**–**H**) are the fitted weights for each period. It is observed that the adjusted parameters of the authority agents indicate a change over time in attitude towards the reduction of economic activities and towards the pandemic. In the case of the population agents, a decrease in compliance with recommendations and a change in attitude towards the reduction of their economic activities in order to reduce the infection rate are observed.

The BTH model has six parameters or weights that determine the attitude of the population agents and authorities towards the infection rate, the economy, and the restrictions (see Supplementary Material). To choose the authorities’ weights, three periods were considered. During the first period (day 0–249), there was significant concern about the infection rate, leading to restrictive measures and a relatively lower priority placed on the economy. In the second period (day 250–499), while most recommendations are maintained, the government starts to give more importance to economy. Finally, in the third period (day 500–1000), after 70% of the population was vaccinated, the infection rate became less of a concern and both the restrictions and the economical contributions made by the government to fight the pandemic, were also reduced (see Fig. 4C–E). The dates of these periods were chosen considering the variation in the curves shown in Fig. 4A,B. Figure 4A shows the stringency index^30^, which is a measure of how strict the government response is to the pandemic. This index ranges from 0 to 100, with 100 being the most restrictive with measures such as school and workplace closures and mobility restrictions, among others. Figure 4 shows the percentage of the population vaccinated with two doses in blue, and the percentage of the population that received vaccine boosters in orange (data obtained from our world in Data^2^) All authority agents’ weights ($W^X_i$, $W^Y_i$, and $W^Z_i$) were considered negative to meet the minimum effort assumptions. Similarly, $w^x_i$ and $w^y_i$ were considered negative. However, it was established that $w^c_i$ should be positive, considering that the general population was initially willing to comply with the restrictions imposed by the authority agents.

In general, the population agents had a more gradual change in behavior. At the beginning of the pandemic, lack of knowledge and need of protection against the disease resulted in a higher level of compliance. However, over time it was observed that the population became increasingly less willing to comply with restrictions and with the arrival of vaccines they sought to recover economic activity. This gradual change is reflected in the model adjustment, dividing time into 6 periods chosen based on the variations in the curves in Fig. 4A,B. The first period had strict restrictions and high compliance (up to 180 days). The second period (181–250 days), coinciding with the summer season, saw lower compliance from the population. The third period (251–500 days) was marked by the emergence of vaccines and fatigue towards restrictions, resulting in reduced compliance from the population and less interest in reducing their own economy. The fourth period (501–600 days) saw accelerated vaccination, motivating the population to return to normality and improve their economical situation. The fifth period (601–800 days) saw the start of vaccine boosters, and for the sixth period most people were fully vaccinated with boosters, reducing the population’s interest in the pandemic significantly and, consequently, their willingness to comply with restrictions or do economical contributions. The behavior mentioned in these periods was reflected in a decrease in the absolute value of the two parameters $w^y_i$, and $w^c_i$ (see Fig. 4G,H) and and increase in the absolute value of the $w^x_i$ parameter.

It should be noted that, as we showed in^17^, the BTH-SEIRS model can return similar results using different sets of value parameters, especially when the ratios of the parameters are the same. In this study we have taken the view that the agents become in time more reluctant towards making pandemic mitigation efforts, but we could also have obtained similar results by assuming that in time the attitudes of the agents tend toward normality, i.e. $W^X_i=W^Y_i=w^x_i=w^y_i=0$, and $W^Z_i$, $w^c_i$ with values considered normal for the modeled society.

### Comparison of numerical simulations with real data

To compare the results of the BTH-SEIRS model to the real world infection rate data and the tourism-based economic proxy, we performed a vast number of simulations with the model and calculated the means of the infection rates and the economic activity. As an error estimate we also evaluated the $90\%$ quantile range calculated by removing $5\%$ of both the highest and lowest values the simulations produced at each time step separately. This form of error estimate has been used in epidemiological contexts and its use is appropriate here due to the highly stochastic nature of the model. As stated above, we treated the SEIRS parameters ($\lambda$, $\epsilon$, etc) as constants that have already been determined by previous works on the pure SEIRS model (see, e.g.^8,9,11^), while we treated the value parameters governing the activities of the agents as adjustable. Our goal was then to find the numerical values of the parameters that produced better match the real world data.

**Figure 5:**
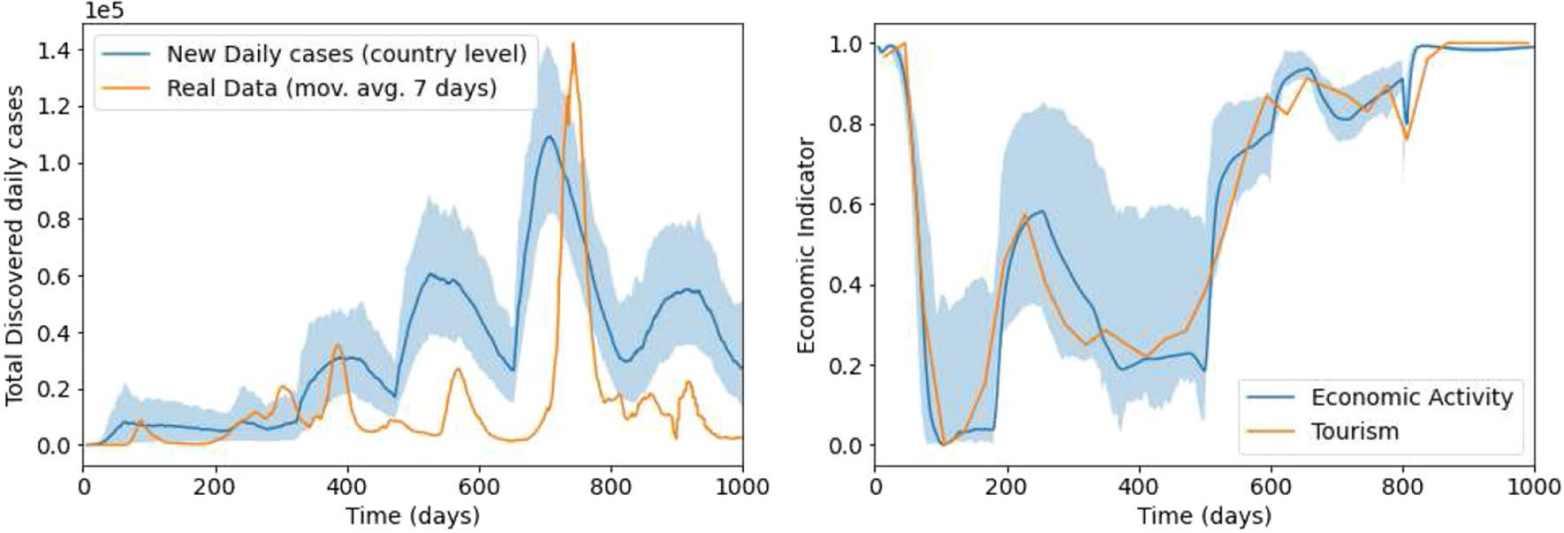
The comparison of the modeled infection rate to the real one (left panel) and the modeled economic activity to the Eurostat tourism data (right panel). The model was run 100 times to obtain the mean, shown as the blue curve. The blue shadow shows the $90\%$ quantile range.

The best fits of the modeled data with the real infection rates and the economic activity levels that we could find can be seen in Fig. 5. The blue shadow represents the area that contains $90\%$ of the iterations. For the fit we prioritised to replicate the economic activity data, which can clearly be seen in the figure. We chose this approach mainly because in this work we wanted to focus on the social side of the BTH-SEIRS model, and secondly because the earlier papers on the SEIRS model have shown that the pure SEIRS model can replicate fairly accurately the infection rates. As a result, the infection rates we get from the model do not usually match quantitatively the real world infection rates, especially in the latter half of the timeline, but at least the peaks of the infection waves are found to occur approximately at the same times. This is to be expected, particularly during the last period of the pandemic, since not all the infected cases that the model presumes were reported in reality. In contrast, we have managed to reproduce the economic activity reasonably well with the exception of the real world peak at around 230 days.

As can be seen from the position of the average simulated economic activity curve in relation to the shadow in Fig. 5, the distribution of the simulated economic activities is not symmetric. At most time steps, the average simulated economic activity is in the lower part of the shadowed area, suggesting a heavy tailed distribution where most of the simulations lie at the lower part of the shadow, while there are also a significant number of outliers in the upper part of the shadow.

## Discussion

In this work, we have shown an application of our previously developed hybrid BTH-SEIRS model that integrates socio-economic and epidemiological behavior to the real case of Covid-19 pandemic in Spain. This model, by its very nature, could be applied to many different situations, so when the BTH weights are fitted, by keeping the biological parameters of the disease fixed and when relating them to the mobility of the populations, that ultimately affect the economic behaviour, one can extract valuable information about the social attitude of the population facing the pandemic. To apply this model, a variable reflecting economic behavior related to activities outside the home, which were most affected by the pandemic, was sought. It was found that tourism, which represents about $10\%$ of Spain’s economic activities, is a suitable indicator.

Once this proxy of economic activity index was established, the model was adjusted considering both social and epidemiological factors. In particular, vaccines and the emergence of new variants, which altered the dynamics of disease transmission, were taken into account. It should be noted that the objective of the work is not to fit the curves of infected individuals, which can be imprecise due to under-reporting of total cases, but to establish a link between these curves and the economic proxy. It is in this sense that the behavior of governmental agents and the population was sought to be established through the weights $W_x$, $W_y$, $W_z$, $w_x$, $w_y$, and $w_c$. Considering the assumptions of the BTH model, it was possible to establish that adjusting the economic proxy was only possible by considering a decrease in the compliance of the population agents with the restrictions imposed by the government. A gradual decrease in interest in the pandemic by both authorities and the population was also considered once vaccination started. Additionally, both population agents and authorities, initially more willing to make economic efforts and impose restrictions to combat the pandemic, gradually changed their attitude in favor of improving the economy. Taking into account the factors considered, it was possible to adjust the model to the economic proxy and appropriately reproduce the appearance of waves of the disease.

The changing attitudes of people and governments toward COVID-19 and the NPIs meant to mitigate its spread have been the focus of intense empirical studies. Most of these have made use of various social media outlets (see e.g.^38,39^ and references therein). While these studies reveal a more detailed picture of the public attitudes than our very simple model, in general they paint a picture similar to one that emerges from our model. That is, they find that the public attitudes towards NPIs had the tendency to become more positive as the COVID-19 epidemic intensified. These studies also report initially sceptical attitudes that are not found in our model, but when one considers the difference in time scales (our simulations covered almost 3 years, but^38,39^ deal with periods of a few month at most at the very beginning of the pandemic), it is clear that including a sceptical period in our model would have made little difference. All in all, the changes in the attitudes of population agents seem to agree qualitatively with the public attitudes deduced from the social media data, at least in the first phase of the pandemic.

The policy responses of the authority agents in our model are driven by their attitudes. Comparing its results with the results of^28^ found that the countries of the European Union with populations of at least 10 million people exhibited three different behavioural types in their response to the first wave of the pandemic in 2020: prepandemic type with no NPIs, deep lockdown, and gradual opening. It was found that as the pandemic progressed, the countries changed their behaviours according to the time evolution of the pandemic, which generally meant moving from the prepandemic state to deep lockdown, after which they tended to move between lockdown and opening states. Spain, in particular, was found to move from the first deep lockdown to a brief relaxation period followed by another lockdown, which was longer than usual in the EU. In our model the authority agents become more willing to relax the epidemic mitigation measures in time, as can be seen in Fig. 4, but the time period we simulate is much longer than just the first wave. It is apparent from the figure that the model is not sensitive enough to pick up the nuances such as those found in^28^.

It should be emphasized that the chosen proxy for economic activity is linked with population activities that have experienced greater variation due to the pandemic and thus depend on the population mobility. In this sense, the adjustment was possible because our hybrid model includes mobility as a fundamental variable linking the socio-economic part with the epidemiological part. The fact that the value parameters can be adjusted in time during the simulations suggests that the BTH-SEIRS model could be improved by dynamically changing them according to a given simulated epidemic situation. For example, making the parameters dependent on the cumulative number of cases is justifiable in the BTH sense, since the agents can be thought to be more afraid of being hit by a personal disaster that only a few of them are affected by, rather than one that is considered “normal”. This is because rare personal disasters lower the social position of an agent relative to most other agents, while “normal” disasters have little effect on the agent’s relative social position. This dynamic value parameter problem is the most important potential line of study for future work. Other ways to improve the model in the future center around strengthening the connection between the modeled agents and their real life counterparts. For example, the modeled agents could be allowed to implement simulated NPIs, rather than using abstract activity reductions, and be allowed to communicate with each other about what kinds of NPIs are appropriate for the epidemic situation.

### Supplementary Information

Supplementary Information.

## Data Availability

The data on coronavirus infections used as part of this project has been taken from the Our World in Data database (https://ourworldindata.org/coronavirus). The Eurostat dataset “Monthly nights spent at tourist accommodation establishments” can be found at https://ec.europa.eu/eurostat/web/products-datasets/-/tour_occ_nim. The programs that run the simulations are available from the authors on reasonable request.
