# Supplementary material for: Socio-economic pandemic modelling: case of Spain
Source: Sci Rep. 2024 Jan 8;14:817. doi: 10.1038/s41598-023-44637-y (PMC10774333; doi:10.1038/s41598-023-44637-y)
Supplement: Supplementary file 1 — Supplementary Information. [file 41598_2023_44637_MOESM1_ESM.pdf]

Supplementary Material

1 Agent-based model features

There are two kinds of agents in our model. Those who represent authorities governing certain region or province (j) in the country, and those representing the normal population of the country. The latter are described by the variables $x_{i,j}$, reduction in the normal economic activity by agent (i) reflecting its effort put to epidemic mitigation , $y_{i,j}$ the proportion of infected people, $c_{i,j}$ the compliance to the measures recommended by the authorities.

In turn, the authorities state variables are denoted by capital letters: $X^{r(j)}$ represents their measures recommended for the economic activity level reductions, $Y^{r(j)}$ are the regional infection rates and the regional reduction in economic activity $Z^{r(j)} = P_{r(j)}^{-1} \sum_{k \in r(j)} z_k$, where $r(j)$ is the region under the administration of authority agent j and $P_{r(j)}^{-1}$ is the number of population agents in the region $r(j)$.

The epidemic mitigation efforts by the population agents are bounded, $x_{i,j} \in [0,1]$ is the reduction in the economic activity of the population agents in an effort to mitigate the spreading of the epidemic, meaning that the compliance of agent i towards the authorities recommendations is just the difference $c_{i,j} = x_{i,j} - X^{r(j)}$, where $X^{r(j)}$ is the recommendation of the authority agent administering the region $r(j)$ population agent i lives in.

The mobility of people v affects the probability that the epidemic spreads between two neighbouring or distant cells, which is assumed to be linearly dependent on $x_{i,j}$. This leads to the relation of the form $v_{i,j}^t = v_0 - x_{i,j} v_{max}$, where $v_{i,j}$ is the mobility causing epidemic to spread from geographical cell i to another cell, v_0 is the mobility of people when no pandemic is present and v_{max} is the maximum obtainable reduction in mobility an agent can afford.

All in all, the motivations of population agents in our model are defined by their efforts to mitigate the epidemic, the infection rate $y_{i,j}$ in their cell, and their compliance, while for the authority agents the corresponding measures are the recommended economic activity level reductions, regional infection rates $Y^{r(j)}$ and the regional reduction in economic activity $Z^{r(j)}$. These concerns give rise to the following BTH utility functions,

$$u_i = w_i^x (x_i + \sum_{k \in e_i} (x_i - x_k)) + w_i^y (y_i + \sum_{k \in e_i} (y_i - y_k)) + w_i^c (c_i + \sum_{k \in e_i} (c_i - c_k)), \quad (1)$$

$$U_i = W_i^X (X_i + \sum_{k \in E_i} (X_i - X_k)) + W_i^Y (Y_i + \sum_{k \in E_i} (Y_i - Y_k)) + W_i^Z (Z_i + \sum_{k \in E_i} (Z_i - Z_k)), \quad (2)$$

where u_i refers to the utility of the population agent i and U_i likewise to the utility of the authority agent i , and we have denoted $x_i = x_{i,j}$,$y_i = y_{i,j}$,$c_i = c_{i,j}$, $X_i = X^{r(i)}$, $Y_i = Y^{r(i)}$,$Z_i = Z^{r(i)}$ for the sake of brevity. $e_{i,j}$ and E_i are the groups of other agents that a given population or authority agent i compares itself with. For the population agents, e_i is the set of their diagonal and adjacent neighbours, while for the authority agents E_i is the set of all other authority agents. The coefficients w_i^x, w_i^y, w_i^c, W_i^X, W_i^Y, and W_i^Z are weights that describe how much the agents value each variable that form the basis of their motivations, so w_i^x indicates how much population agent i values mitigating the epidemic by reducing economic activity and W_i^Z how much authority agent i values regional economic activity, for example. As such, we call these weights value parameters.

It should be noted that the sign of a value parameter determines whether an agent has a positive or negative attitude toward the variable associated with the parameter. Thus $w_i^c > 0$ means that the population agent i regards compliance towards authorities' recommendations positively, and $w_i^c < 0$ the opposite. This has implications for the value ranges the value parameters can have considering the way we derive the actual economic behaviour of the agents. We assume that the agents will generally make minimum efforts to mitigate the epidemic allowed by their respective utility functions (i.e. we assume that $u_i = U_i = 0$ for all agents), which leads to following relations

$$x_i = \frac{1}{|e_i|+1} \left(\frac{w_i^x}{w_i^x + w_i^c} \sum_{k \in e_i} x_k - \frac{w_i^y}{w_i^x + w_i^c} (y_i + \sum_{k \in e_i} (y_i - y_k)) + \frac{w_i^c}{w_i^x + w_i^c} ((|e_i|+1) X_i^r - \sum_{k \in e_i} c_k) \right), \quad (3)$$

$$X_i = \frac{1}{|E_i|+1} \left(\sum_{k \in E_i} X_k - \frac{W_i^Y}{W_i^X} (Y_i + \sum_{k \in E_i} (Y_i - Y_k)) - \frac{W_i^Z}{W_i^X} (Z_i + \sum_{k \in E_i} (Z_i - Z_k)) \right) \quad (4)$$

that govern the actual reduction in the economic activity by the population agents and the restrictions recommended by the authority agents. This minimum effort assumption means that the value parameters w_i^x, w_i^y, W_i^X and W_i^Y are always negative, while w_i^c and W_i^Z can in principle have both signs, even though it may be somewhat counterintuitive in the case of the former, as positive W^Z means that the authority agents actually like having the economic activity in their regions reduced.

2 SEIRS model features

The SEIRS dynamics of each geographic cell is governed by the following set of parameters:

ε: the period of latency before those who contract the disease become infectious

σ: the period of infectiousness

ω: the period of immunity

β_k: the disease transmissivity or transmission coefficient of the $k-th$ variant

μ: the mortality rate

λ: the portion of the population that is susceptible again after being recovered i.e. those who have not succumbed to the infection.

ξ: the effective immunization rate

Time evolution of the numbers of susceptible (S), exposed (E), infected (I), recovered (R), and vaccinated (V) are governed by the following set of difference equations:

$$\begin{aligned} S_{t+1} &= q(S_t - G_t + \lambda q^b G_{t-b} - \xi S_t) + \mu N \\ E_{t+1} &= q(E_t + G_t - q^\varepsilon G_{t-\varepsilon}) \\ I_{t+1} &= q(I_t + q^\varepsilon G_{t-\varepsilon} - q^a G_{t-a}) \\ R_{t+1} &= q(R_t + q^a G_{t-a} - q^b G_{t-b}) \\ V_{t+1} &= q(V_t + \xi S_t), \end{aligned} \tag{5}$$

where we denote $q = (1-\mu)$, $a = (\varepsilon + \sigma)$, $b = (\varepsilon + \sigma + \omega)$ and $G_t = S_t(1 - e^{-\beta_k I_t})$. Whenever the epidemic spreads to a cell, the initial amount of infected is given by the parameter η, such that $S_{t_0} = 1 - \eta$ and $I_{t_0} = \eta$ at the time t_0 of spreading.

As seen in the equations, this model takes into account vaccination. This is done simply by including a compartment (V) where individuals enter under two conditions: if they have acquired sterilizing immunity, meaning they are no longer susceptible to infection, or if, upon becoming sick, they develop the disease with low virulence and therefore are not contagious. However, it has been observed that only a percentage of the population reaches this condition and that it is lower than the actual number of vaccinated individuals. In this sense, an effective immunization rate ξ lower than the actual vaccination rate is used.

The spread of the virus at the macro-scale, between basic geographic units, occurs through 3 mechanisms: spread to neighboring cells, spread to distant cells, and "Thermal Noise". The latter represents the random movements of people to places not directly connected. The first two mechanisms of propagation depend on Monte Carlo algorithms that compare the parameter ν, which represents mobility, with a random value and initiate a new disease outbreak in the $i-th$ cell so that $S(i)_{t_0} = 1 - \eta$ and $I(i)_{t_0} = \eta$. The difference between these two mechanisms is that, in the case of long-distance spread, there must be a transit connection (by land, rail, or air) between both cells. The relative traffic among cells is taken into account using the parameter ν weighted by the product of normalized population densities of the origin and destination cells.

To consider the "thermal noise", which reflects the randomness of population mobility, a random number is compared with the expression $exp(-1/KT)$ to initiate an outbreak in the $i-th$ cell in the same way as before. In this case, the basic unit i is randomly chosen from those with low population density. To account for cases where government restrictions prevent mobility of people within basic geographic units, the "local stochastic" propagation mechanism was incorporated. This mechanism also depends on ν and allows sick people to continue with the evolution of their disease according to equations 5, but prevents the appearance of new cases if a random number is greater than ν. For more details on the mentioned propagation mechanisms, see articles by Barrio et al.[1,2] and Barreiro et al.[3].

The variants were incorporated into the model in a simple way, taking into account the work done by Barreiro et al.[4]. In this work, it is considered that each variant differs from others by having its own transmission coefficient β_k which dominates the dynamics of contagion of the specific strain. Considering that it has been observed that each new variant of concern dominates over the previous ones, reducing their circulation[5], we will regard that only the most contagious variant is propagated, ignoring the others. In this way β_k is changed every time a new strain (k) appears. The β_k values used for each variant were obtained from Barreiro et al.[4]

References

1. Barrio, R., Varea, C., Govezensky, T. & José, M. Modeling the geographical spread of influenza A(H1N1): The Case of Mexico. Appl. Math. Sci. **7**, 2143–2176, DOI: 10.12988/ams.2013.13193 (2013).
2. Barrio, R. A., Kaski, K. K., Haraldsson, G. G., Aspelund, T. & Govezensky, T. Modelling Covid-19 epidemic in Mexico, Finland and Iceland. Phys. A **582**, 126274 (2021).
3. Barreiro, N. L., Govezensky, T., Bolcatto, P. G. & Barrio, R. A. Detecting infected asymptomatic cases in a stochastic model for spread of Covid-19. The case of Argentina. Sci. Reports **11**, 10024, DOI: https://doi.org/10.1038/s41598-021-89517-5 (2021).
4. Barreiro, N. L. et al. Modelling the interplay of SARS-CoV-2 variants in the United Kingdom. Sci. Reports 12372, DOI: https://doi.org/10.1038/s41598-022-16147-w (2022).
5. Hodcroft, E. B. Covariants: SARS-CoV-2 mutations and variants of interest (2021). https://covariants.org/.
